# Plant microbiome engineering: from inoculation to genome editing

**DOI:** 10.3389/fmicb.2026.1781381

**Published:** 2026-04-22

**Authors:** Jyoti Yadav, Pushpa Gehlot, Priya Soni, Tripta Jain

**Affiliations:** Microbial Research Laboratory, Department of Botany, Mohanlal Sukhadia University, Udaipur, India

**Keywords:** CRISPR, holobiont, plant microbiome, sustainable agriculture, synthetic biology

## Abstract

Plant-associated microbiomes are central to crop productivity, nutrient efficiency, and stress resilience, yet conventional microbiome manipulation strategies, largely based on microbial inoculation and agronomic management, often suffer from inconsistent field performance and limited persistence. Although several recent reviews have discussed CRISPR-mediated plant–microbe engineering and synthetic microbial community (SynCom) design separately, few reviews integrate genome editing, ecological stability of microbiomes, and climate-resilient agricultural applications within a unified conceptual framework. Recent advances in molecular biotechnology are transforming this landscape by enabling precision engineering of plant-microbe interactions at genetic, metabolic, and community levels. In particular, synthetic biology tools including CRISPR/Cas genome editing, RNA interference, and synthetic microbial communities (SynComs), now allow targeted modification of plant traits governing microbial recruitment, microbial pathways underpinning nutrient cycling and stress tolerance, and community-level functional complementarity. This review integrates molecular genetics, microbial ecology, and systems-level microbiome design to frame the plant and its microbiome as an engineerable holobiont. We integrate insights from genome editing in plants and microbes, omics-guided SynCom design, climate-resilience mechanisms, and emerging AI-assisted decision frameworks, including machine learning and ecological modeling approaches used to analyze multi-omics datasets, and predict plant–microbiome interactions across experimental and field-based studies. Importantly, we critically assess limitations related to ecological stability, trait trade-offs, biosafety, and regulatory challenges that constrain large-scale deployment. By bridging genome-enabled microbiome manipulation with ecological design principles, this review proposes an integrative framework for climate-smart microbiome engineering and identifies key research priorities required to transition from empirical inoculation toward predictive, sustainable, and socially responsible agricultural biotechnology.

## 1 Introduction

Plant-associated microbiomes, especially the rhizosphere community that surrounds roots, are now recognized as essential determinants of agricultural productivity, abiotic-stress tolerance, and long-term ecosystem sustainability (Mukherjee et al., 2024; Rosete-Enríquez et al., 2025). Microbes supply nitrogen, solubilize phosphorus, produce phytohormones and antimicrobial compounds, and prime host immunity, thereby allowing crops to maintain yields while reducing synthetic fertilizer and pesticide inputs (e.g., bio-fertilizer applications have increased wheat grain yield by ≈ 12% under low-N conditions) (Afridi et al., 2022). The concept of the plant-microbiota holobiont has reshaped our understanding of plant health. Although the status of the holobiont as a strict evolutionary unit remains debated, the framework is widely used in a functional and ecological sense to describe the integrated interactions between host plants and their associated microbial communities that influence plant performance and ecosystem processes (core and hub taxa such as *Pseudomonas* or *Bacillus* act as keystone nodes that stabilize community function) (Qiu et al., 2019; Dini-Andreote et al., 2015). Historically, rhizosphere engineering has relied on inoculation-centric practices. Seed bio-priming or coating with plant-growth-promoting rhizobacteria (PGPR) improves early root colonization and can boost seedling vigor, yet the benefits often diminish after storage or under field stresses (Yang et al., 2024). Direct soil drenching of single-strain inoculants (e.g., *Bacillus* spp.) has yielded modest yield gains but suffers from rapid population loss ( > 80% within weeks) because introduced microbes are outcompeted by the native resident microbiota (O’Callaghan et al., 2022). More holistic agronomic tactics, such as organic amendments, crop rotation, and reduced tillage, modify the physicochemical environment to favor beneficial assemblages; for instance, integrating sweet-potato and legume rotations increased microbial diversity and raised overall farm productivity by ≈ 38% while cutting N_2_O emissions by ≈ 39% (Joshi et al., 2025). Synthetic consortia assembled from multiple isolates can outperform single strains: a two-species *Pseudomonas*/*Bacillus* cocktail enhanced maize biomass by 27.8% in field trials (De la Vega-Camarillo et al., 2023). Despite these successes, conventional methods are limited by their reliance on exogenous inputs, variable establishment rates, and an inability to steer host-mediated recruitment signals.

**FIGURE 1:**
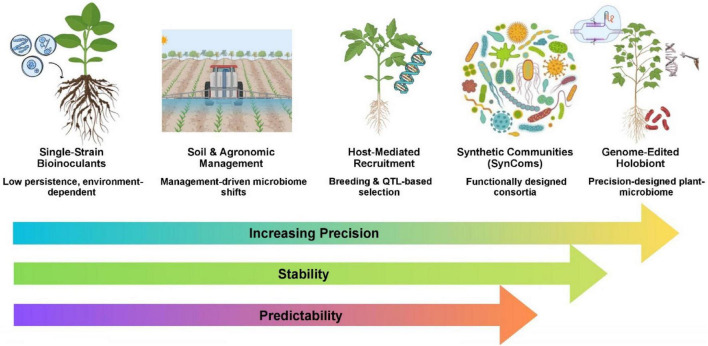
Conceptual overview of plant microbiome engineering approaches, ranging from single-strain bioinoculants to genome-edited holobionts. The progression highlights increasing precision, stability, and predictability of plant-microbiome interactions through agronomic management, host-mediated recruitment, synthetic communities, and genome-enabled microbiome design.

The advent of genome-editing technologies offers a complementary, host-directed route to microbiome engineering (Amen et al., 2025). CRISPR-Cas9 enables precise alteration of plant genes that shape root architecture, exudate composition, and immune signaling, thereby influencing microbial recruitment (Ansori et al., 2023). Knock-out of root-hair regulators in barley reduced rhizosphere α-diversity, confirming a causal link between root morphology and community assembly (Robertson-Albertyn et al., 2017). In maize, loss-of-function mutations that suppress benzoxazinoid production dramatically reshaped bacterial and fungal communities, conferring transgenerational herbivore resistance, even after a winter fallow (Kudjordjie et al., 2019). QTL mapping of mycorrhizal responsiveness in legumes has identified host loci that can be edited to strengthen symbiotic phosphorus acquisition (a strategy now being translated to cereals) (Li et al., 2023). Moreover, synthetic community (SynCom) approaches, which are defined by design and functionally complementary microbial assemblages, allow researchers to test and deploy microbiomes with predictable traits. This is demonstrated by a 10-strain SynCom that increased maize shoot dry weight by approximately 28% under field conditions (Shayanthan et al., 2022). Viewing the plant and its associated microbes as an integrated holobiont suggests that the most durable and climate-resilient solutions will combine host genetic improvement with rationally designed microbial partners. Climate-smart agriculture demands that engineered holobionts not only sustain yields under drought, salinity, or temperature extremes but also minimize environmental footprints (Portal-Gonzalez et al., 2025). Figure 1 summarizes the conceptual progression of plant microbiome engineering. Recent work emphasizes that microbiome-guided breeding can reduce fertilizer demand, lower greenhouse gas emissions, and improve water-use efficiency, aligning with climate-smart goals. At the same time, responsible innovation frameworks are crucial for addressing biosafety, regulatory, and societal concerns associated with genome-edited crops and genetically modified microbes. In parallel, advances in synthetic biology now enable the programmable design of plant-associated microbes through modular genetic circuits, engineered metabolic pathways, and microbial chassis optimized for rhizosphere functions.

This review aims to synthesize recent advances in plant microbiome engineering by integrating insights from molecular genetics, microbial ecology, and agricultural biotechnology. Specifically, the article addresses three key questions: (i) how genome-editing technologies such as CRISPR/Cas can be used to manipulate plant traits that influence microbiome recruitment and function, (ii) how synthetic microbial communities (SynComs) can be rationally designed to enhance nutrient acquisition, stress tolerance, and disease suppression, and (iii) how emerging tools including multi-omics, artificial intelligence, and digital agriculture platforms can improve the predictability and field stability of engineered microbiomes.

**FIGURE 2:**
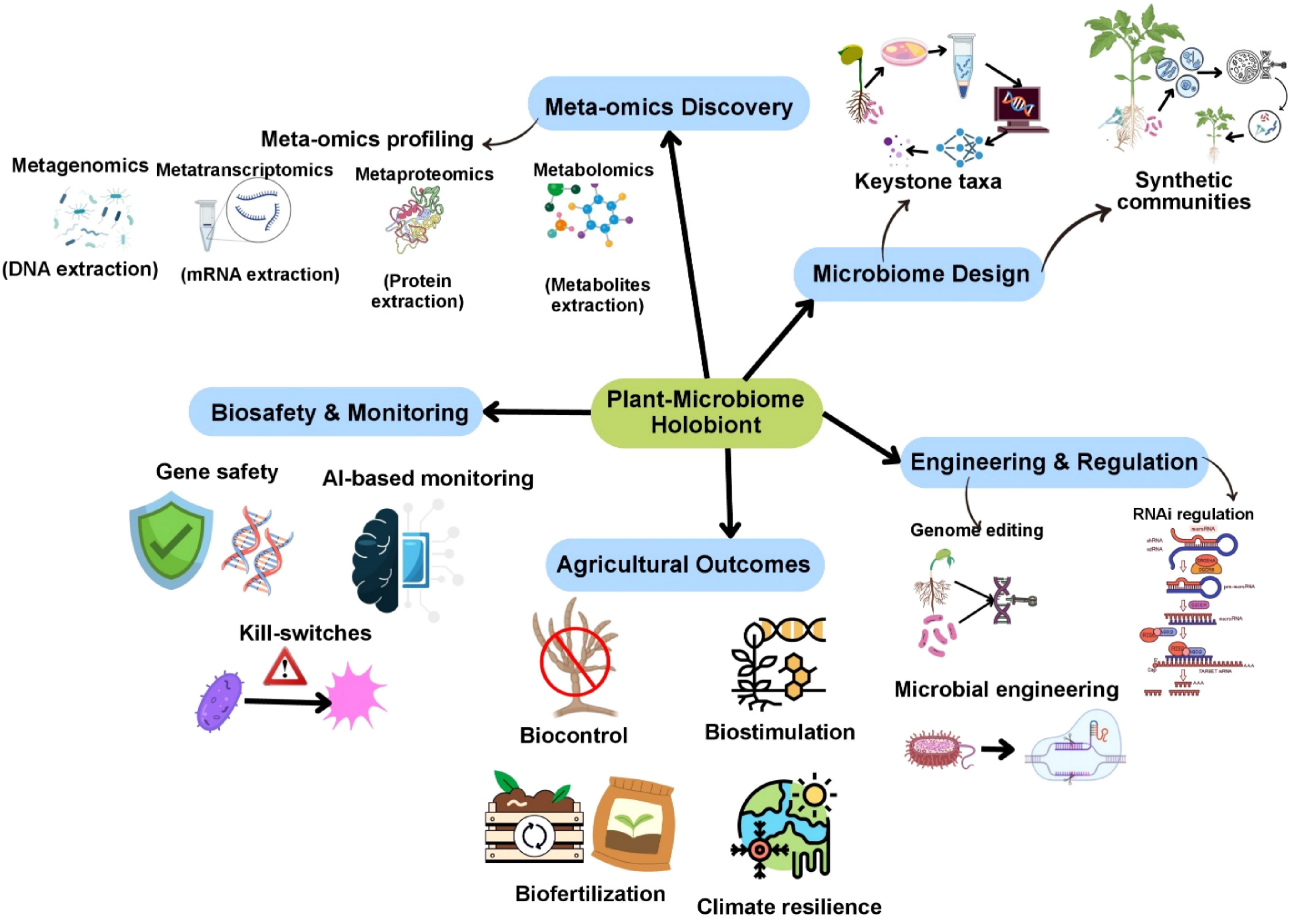
Integrated framework for plant-microbiome holobiont engineering. Meta-omics-driven discovery informs microbiome design, synthetic community assembly, and genome-enabled engineering, supported by biosafety and monitoring strategies to achieve improved agricultural productivity and climate resilience.

Unlike previous reviews that discuss genome editing, SynCom design, synthetic biology approaches, climate-resilient agriculture, and digital decision-support systems as separate developments, this article integrates these approaches within a holobiont-centric framework in which plant genomes, microbial consortia, and environmental feedback are co-engineered. Figure 2 presents an integrated framework for holobiont engineering of the plant–microbiome. By synthesizing molecular mechanisms, multi-omics insights, field-scale evidence, and biosafety considerations, the review highlights how microbiome engineering can transition from empirical inoculation practices toward predictive, climate-smart, and evolution-aware agricultural biotechnology.

## 2 Conventional approaches to microbiome manipulation

Manipulation of the plant-associated microbiome has traditionally relied on three main approaches: direct inoculation with beneficial microbes, indirect recruitment through plant breeding and engineering, and modification of soil environments via agricultural practices (Ali et al., 2024). Each strategy has advanced our understanding of plant-microbe interactions and provided practical tools for crop production; yet, their successes and limitations highlight the complexity of stabilizing microbiomes in open-field conditions. Table 1 compares conventional plant microbiome manipulation strategies.

**TABLE 1:** Comparison of traditional microbiome manipulation methods (approach, advantages, limitations).

Strategy	Mechanism	Representative case study	Advantage	Limitation	Reference
Seed-coat and root-dip inoculation	Direct delivery of selected bacteria/fungi onto the germinating seed or seedling roots to ensure early colonization of the rhizosphere.	•*Pseudomonas fluorescens* seed-coating increased wheat grain yield by 7% under low-N field conditions in the Turkey. •Wheat seeds coated with *Bacillus* spp. showed a 15% increase in seedling vigor and reduced *Fusarium* incidence Saudi Arabia.	•Guarantees early root colonization •Low inoculum loss compared with soil-drip •Simple to integrate into existing sowing equipment	•Viability can decline during storage and transport •Limited control over spatial distribution after germination •May not persist under harsh field conditions	(Moussa et al., 2013; Erdemci et al., 2019)
Soil-borne bio-fertilizers (e.g., mycorrhizal inoculants, rhizobia)	Introduction of symbiotic microorganisms that enhance nutrient acquisition or nitrogen fixation	•Arbuscular mycorrhizal (AM) inoculation significantly enhances phosphorus uptake and fertilizer efficiency, enabling up to 25–100% yield increases and 44% higher P-use efficiency while allowing reduced fertilizer inputs	•Direct introduction of functional microbes (N-fixers, P-solubilizers, biocontrol agents) •Well-established commercial products	•Rapid decline of introduced populations due to competition with indigenous microbiota •Variable efficacy across soils, climates, and cropping systems •Risk of non-target effects if the strain is not well-adapted	(Hao et al., 2014; Cabrales et al., 2019; Qian et al., 2024)
Organic amendments (compost, manure, biochar)	Supply of organic carbon and micronutrients that favor beneficial microbes while suppressing pathogens.	•Compost and *Bacillus*-based interventions markedly suppress rice sheath blight, reducing disease severity by 32.6–39.9% with compost and up to 64.7% with *Bacillus* strains	•Supplies carbon and nutrients that stimulate native beneficial microbes •Improves soil structure, water-holding capacity and overall fertility •Reduces reliance on synthetic chemicals	•Effects are indirect and depend on amendment quality and application rate •May also stimulate opportunistic pathogens if not properly composted •Nutrient release can be slow, requiring careful timing	(Nuryanto et al., 2023; Krishnan et al., 2025)
Crop rotation and inter-cropping	Alternating host plants to reshape the rhizosphere community and break disease cycles.	•A wheat-legume rotation in the Canadian Prairies increased the relative abundance of disease-suppressive *Streptomyces* spp. and lowered *Fusarium* head-blight severity. •Wheat-peanut rotation in the North China Plain increased rhizosphere microbial diversity and raised crop yield by 38% while cutting N_2_O emissions by 39%	•Alters the bulk-soil microbial pool, favoring disease-suppressive taxa •Breaks pathogen life cycles and reduces inoculum pressure •Enhances nutrient cycling (e.g., N-fixation by legumes)	•Requires long-term planning and compatible market demand •Benefits may be modest if rotation crops share similar root exudate profiles	(Woo et al., 2022; Yang et al., 2024)
Soil physicochemical management (tillage, irrigation, pH adjustment)	Modifying the abiotic environment to select for desired microbial guilds.	•Reduced tillage in a Californian almond orchard raised the diversity of Actinobacteria and correlated with improved drought resilience. •Biochar application in soybean systems improves yields by 4.7–6.4% on average and up to 28–37% under favorable soil and climatic conditions	•Preserves soil aggregates and the existing microbial habitat •Encourages growth of mycorrhizal fungi and other slow-growing taxa •Reduces erosion and fuel use	•Can lead to stratification of nutrients and compaction if not combined with cover crops •Weed pressure may increase, requiring additional control measures	(Yooyen et al., 2015; Dokoohaki et al., 2019; Fenster et al., 2021; Conde et al., 2024)
Biopesticide/ biostimulant sprays	Foliar or drench applications of microbial metabolites or live cells that trigger induced systemic resistance.	A *Bacillus subtilis* based biopesticide applied to tomato reduced bacterial spot disease by 48%.	•Induce systemic resistance (ISR) and can suppress specific pathogens •Can be applied as foliar or soil drenches, offering flexibility	•Effectiveness is highly dependent on timing, pathogen pressure, and environmental conditions •Repeated applications may be needed, increasing cost	(Naue et al., 2014)

### 2.1 Inoculation with beneficial microbes

Direct inoculation with plant growth-promoting rhizobacteria (PGPR) and fungi (PGPF) has been widely explored to enhance nutrient acquisition, stress tolerance, and pathogen resistance (Khoso et al., 2024). These inoculants often function through mechanisms such as nitrogen fixation, phosphorus solubilization, hormone production, or siderophore-mediated iron acquisition (Shahwar et al., 2023). While greenhouse studies frequently report strong growth-promoting and disease-suppressive effects, field translation has been inconsistent. The major challenges include competition with native soil microbiota, sensitivity to environmental fluctuations, and difficulties in achieving long-term establishment. For instance, several microbial inoculants that performed effectively under controlled conditions failed to persist in agricultural soils once exposed to diverse indigenous communities and fluctuating abiotic stresses (Mawarda et al., 2020). Nevertheless, some success stories exist: rhizobacterial inoculation has been shown to enhance micronutrient biofortification in cereals, while specific consortia have improved root colonization and induced systemic resistance (Sharma et al., 2025). These examples underscore the promise of inoculation but also point to the need for integrating ecological and genetic considerations to improve field stability.

### 2.2 Plant breeding and engineering

An alternative to direct inoculation is leveraging the host plant’s genetic capacity to recruit and regulate its microbiome. Decades of research demonstrate that plant genotype significantly shapes microbial assembly, from root exudation patterns to immune signaling pathways. However, modern breeding and domestication, which prioritized yield under high-input systems, often eroded the genetic traits needed to sustain beneficial microbial partnerships (Bouwmeester et al., 2025). A well-documented case is that of broad bean domestication, in which cultivated varieties retained fewer rhizobial partners than wild progenitors (Liu A. et al., 2020). Similarly, soybean breeding reduced the plant’s ability to exclude ineffective rhizobia, leading to inefficient symbioses (Liu J. et al., 2020; Pérez-Jaramillo et al., 2015). Recent advances in molecular breeding and genome editing open new opportunities to restore or enhance these interactions. Traits such as root hair density, cuticle permeability, or secondary metabolite production have been linked to shifts in microbial recruitment (Du et al., 2025). For example, maize mutants deficient in benzoxazinoid production dramatically altered rhizosphere community composition and affected the performance of subsequent plant generations, even after fallow periods (Kudjordjie et al., 2019). Perhaps the most striking example is a Mexican maize landrace that secretes sugar-rich mucilage from aerial roots, fostering nitrogen-fixing microbial consortia and reducing reliance on chemical fertilizers (Gao et al., 2023). Such cases illustrate the potential of breeding and genetic engineering not only to restore lost symbioses but also to design novel plant traits that stabilize beneficial microbiomes under field conditions.

### 2.3 Agricultural practice-based strategies

Conventional agricultural practices exert strong and predictable control over soil microbial community structure by reshaping nutrient availability, ecological interactions, and microbial network topology, thereby indirectly governing the assembly of plant-associated microbiomes (Fadiji et al., 2025). Crop rotation, organic amendments, intercropping, conservation tillage, and agroforestry modify soil carbon inputs, nutrient stoichiometry, and physical structure, creating selective pressures that favor distinct microbial functional guilds. Legume-based rotations enrich soils with organic acids that mobilize phosphorus, enhancing nutrient availability for subsequent cereal crops (Al-Shammary et al., 2024). Green manuring with sunn hemp (*Crotalaria juncea*) and dhaincha (*Sesbania aculeata*) significantly increases microbial activity and soil organic matter, translating into sugarcane yield gains of up to 57% (Bokhtiar et al., 2003). Conservation tillage and residue retention promote microbial biomass, stabilize fungal hyphal networks, improve soil aggregation, and enhance nutrient retention, while agroforestry systems contribute to long-term soil fertility and resilience under climatic stress (Fatima and Ying, 2025). Evidence from long-term field experiments highlights the dominant role of fertility inputs in structuring microbial communities. The Farming Systems Trial at the Rodale Institute demonstrated that the fertility source, synthetic fertilizer, legumes, or manure, was the strongest determinant of microbial community composition, with fungi particularly responsive to organic inputs (Longenecker, 2022). Machine learning analyses resolved discrete microbial co-occurrence modules linked to fertility regimes: synthetic fertilization favored bacterial hub taxa, whereas legume-based fertility promoted fungi-dominated networks, with effects detectable to depths of 30 cm (Mo et al., 2024). These findings indicate that conventional management practices, particularly fertilizer choice, not only sustain soil health but also reorganize microbial interaction networks, thereby influencing crop productivity and resilience.

Across diverse cropping systems, disease suppression consistently emerged as the primary microbiome-mediated ecosystem service (Singh et al., 2025). Quantitative outcomes demonstrated substantial reductions in disease incidence across multiple cropping systems. For instance, application of antagonistic *Pseudomonas* consortia suppressed banana *Fusarium* wilt by approximately 39.4% (Lv et al., 2023), while Abdelghany et al. (2024) reported even more pronounced effects in sugar beet, where *Streptomyces* strains reduced disease severity by up to 94.77% (Abdelghany et al., 2024). In tomato, suppressive soils reduced *Ralstonia solanacearum* infection from 86.67% in conducive soils to 53.33% (Zhang et al., 2022), while in wheat, *Bipolaris sorokiniana* severity progressively declined after five successive growth cycles, indicating adaptive restructuring of the microbial community (Costa et al., 2023). Collectively, these results demonstrate that disease suppression is an emergent, time-dependent property of soil microbiomes shaped by repeated plant-pathogen interactions rather than static soil physicochemical traits.

Taxonomic analyses revealed recurrent enrichment of specific bacterial lineages associated with suppressive phenotypes. Although Proteobacteria were often depleted at the phylum level, functionally important members within Myxococcales, Pseudomonadales, Xanthomonadales, and particularly the genus *Pseudomonas* were consistently enriched in suppressive banana and sugar beet soils (Carrión et al., 2019; Shen et al., 2022). *Pseudomonas* spp. are prolific producers of siderophores, phenazines, and polyketides that inhibit pathogens through iron sequestration and antibiotic activity. Actinobacteria were universally enriched across suppressive systems; genera such as *Kribbella* and *Nocardioides* exhibited experimentally validated antagonism against *Pseudomonas syringae* in *Arabidopsis* (Zhang et al., 2022). Firmicutes, particularly *Bacillus*, contributed via antimicrobial peptide production and induction of host defense responses (Jordan et al., 2023). Members of the Bacteroidetes family Chitinophagaceae were repeatedly detected in wheat and sugar beet systems, producing chitinases, glycoside hydrolases, and related enzymes involved in the degradation of fungal cell walls (Carrión et al., 2019; Costa et al., 2023). Enrichment of Chitinophagaceae in rhizosphere and endosphere compartments was negatively correlated with disease incidence and positively associated with activation of plant phenylpropanoid biosynthesis genes (Lai et al., 2025). Additional families, including Comamonadaceae, Anaerolineaceae, Nitrosomonadaceae, and Flavobacteriaceae, were enriched in resistant wheat and sugar beet cultivars, indicating convergence of suppressive functions across phylogenetically distinct taxa (Costa et al., 2023).

The ecological context strongly modulated these patterns. The microbial composition and function varied among bulk soil, rhizosphere, and endosphere compartments, with endosphere communities being more tightly linked to direct pathogen antagonism and rhizosphere taxa being more strongly associated with immune signaling (Costa et al., 2023; Bouwmeester et al., 2025). Temporal dynamics were critical: in wheat, five consecutive growth cycles were required for suppressive microbiomes to fully establish, coinciding with progressive accumulation of BGCs (Chen et al., 2022). Conversely, resistant cultivars exhibited early suppression that gradually diminished over time, indicating reduced microbial selection pressure when host genetic resistance is strong. Large-scale geographic surveys across China (∼300,000 km^2^) and Japan (2,903 sites) confirmed that while specific taxa varied regionally, functional outcomes such as disease suppression and nutrient cycling were conserved (Fan et al., 2020; Fujita et al., 2024), consistent with functional redundancy and convergent evolution in soil microbiomes (Jiao et al., 2022). Archaea, including ammonium-oxidizing Nitrososphaeraceae, contributed indirectly by enhancing nitrogen cycling in low-disease soils (Rodriguez et al., 2021). Fungal taxa such as *Trichoderma* and *Lecythophora* were positively associated with disease suppression and soil fertility in wheat and sugar beet systems (Carrión et al., 2019; Fan et al., 2020; Pastor et al., 2023).

Despite advances, conventional microbiome manipulation strategies remain limited in field scalability and reliability. Microbial inoculants often perform inconsistently due to competition with native microbiota, environmental variability, and poor rhizosphere persistence (Etesami, 2025; O’Callaghan et al., 2022). Likewise, breeding for yield under high-input systems has reduced plant traits supporting beneficial microbial associations (Clouse and Wagner, 2021). Although practices such as crop rotation, organic amendments, and conservation tillage can enhance soil microbial diversity, their effectiveness varies with soil type, climate, and management (Xu et al., 2023; Kovács et al., 2023). These challenges underscore the need for more precise, systems-based microbiome engineering strategies to stabilize plant–microbiome interactions.

## 3 Emerging biotechnological tools for microbiome engineering

Recent advances in microbiome engineering are closely linked to developments in synthetic biology to improve agricultural performance. Synthetic biology approaches facilitate the construction of programmable genetic circuits, the engineering of microbial chassis with optimized metabolic pathways, and the development of biosensors capable of detecting plant stress signals or soil nutrient conditions. These technologies provide powerful tools for designing microbial strains and synthetic communities with predictable functions, thereby expanding microbiome engineering beyond empirical inoculation strategies toward programmable, responsive plant–microbe systems.

### 3.1 CRISPR/Cas genome editing

**FIGURE 3:**
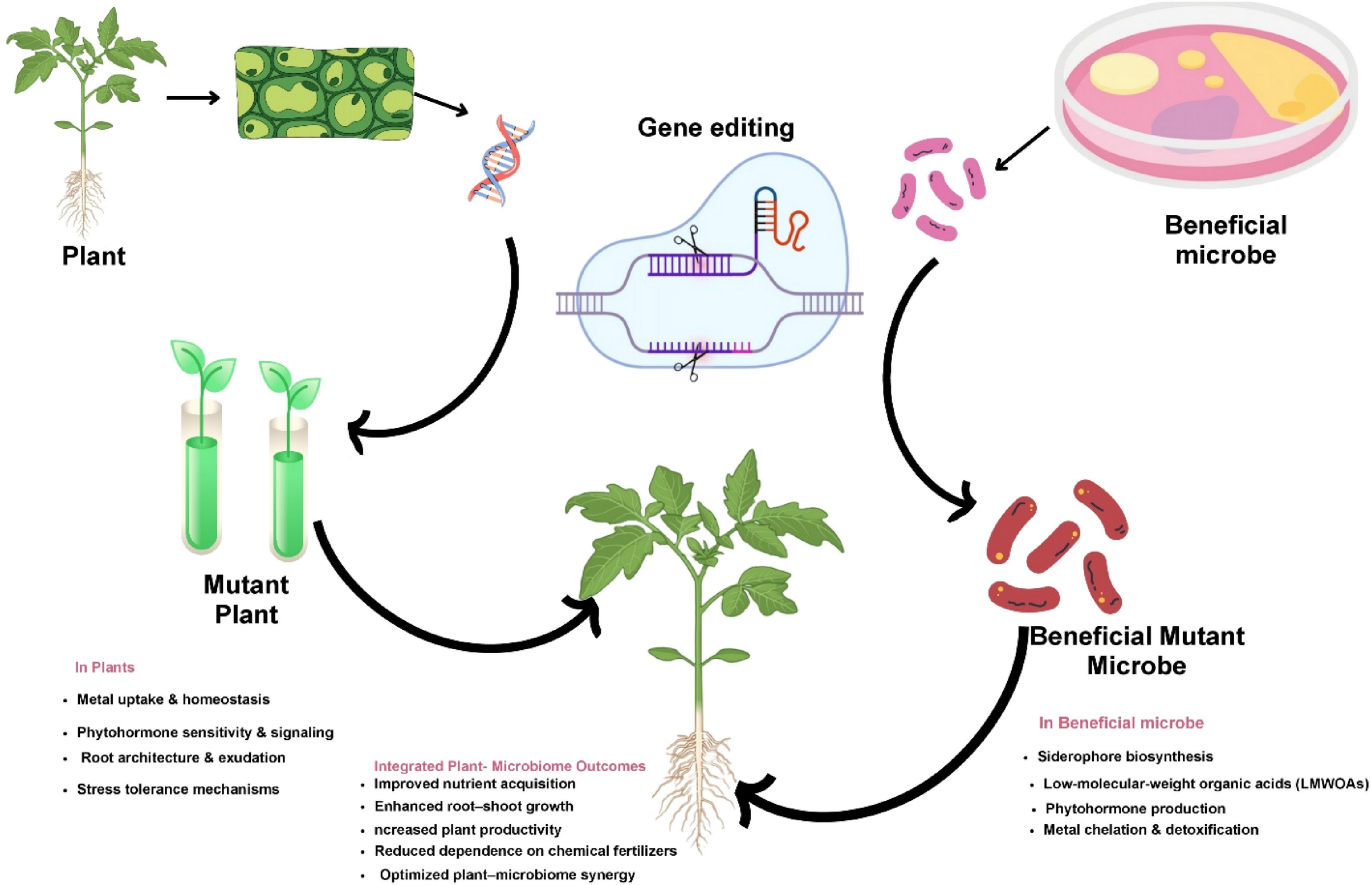
Conceptual overview of CRISPR/Cas-based co-engineering of plants and beneficial microbes. Targeted genome editing enhances plant traits and microbial functions, resulting in improved nutrient acquisition, growth, productivity, and sustainable plant–microbiome interactions.

CRISPR/Cas genome editing has emerged as a powerful tool for microbiome engineering, enabling precise modification of microbial genomes and plant traits that influence rhizosphere interactions (Amen et al., 2025). As illustrated in Figure 3, CRISPR-based co-engineering of plants and beneficial microbes can modify microbial metabolic pathways and plant signaling networks that regulate nutrient acquisition, stress tolerance, and disease resistance in plant–microbiome systems (Sahoo et al., 2025; El-Saadony et al., 2024).

In beneficial rhizobacteria, CRISPR editing has clarified the functional roles of metabolic pathways underlying plant growth promotion. For example, CRISPR-Cas9 mutagenesis in *Bacillus subtilis* HS3 and *Bacillus mycoides* EC18 demonstrated that disruption of siderophore biosynthesis genes, such as *dhbB*, abolished bacillibactin production and significantly reduced root colonization and growth promotion in *Brassica rapa* (Yi et al., 2018). Similarly, knockout of the *sfp* gene in *B. subtilis* impaired the synthesis of lipopeptides including surfactin and fengycin, leading to reduced antifungal activity against soilborne pathogens. These studies highlight the importance of siderophore and lipopeptide biosynthesis pathways in rhizosphere colonization and microbial biocontrol.

CRISPR editing has also been applied to symbiotic fungi to selectively modify metabolite production. In the endophytic fungus *Epichloë* sp. LpTG-3 strain AR37, deletion of indole-diterpene biosynthetic genes eliminated the production of epoxyjanthitrems associated with livestock toxicity while maintaining the symbiotic association with host grasses (Miller et al., 2022). Such targeted modifications illustrate how genome editing can optimize beneficial plant–microbe interactions without disrupting symbiosis. Genome editing in plants can further influence rhizosphere microbiomes by altering root architecture and exudate composition that regulate microbial recruitment. For instance, disruption of the *MAX1* gene in tomato reduced the exudation of the strigolactone orobanchol and altered rhizosphere signaling processes (Bari et al., 2021). Similarly, editing of *CCD7* in rice reduced strigolactone production and modified root-mediated signaling associated with plant–microbe interactions (Butt et al., 2018).

Recent advances increasingly integrate CRISPR editing of both plants and microbes to design optimized plant–microbiome systems. Synthetic biology approaches are being used to engineer microbial strains and consortia with enhanced nutrient acquisition, stress tolerance, and disease suppression capacities (Iqbal et al., 2023; Patyal et al., 2025). Examples include CRISPR-engineered nitrogen-fixing bacteria that improve wheat productivity and modified *Pseudomonas* strains that enhance phosphate availability in rice (Chen et al., 2024). These co-engineering strategies illustrate the potential of genome editing to enable targeted manipulation of plant–microbiome interactions for sustainable agriculture.

Despite these advances, challenges remain, including off-target effects, ecological stability of engineered microbes, and biosafety considerations. Continued improvements in genome-editing precision and regulatory frameworks will therefore be essential for the responsible deployment of CRISPR-based microbiome engineering in agricultural systems.

### 3.2 RNAi and advanced molecular approaches

**FIGURE 4:**
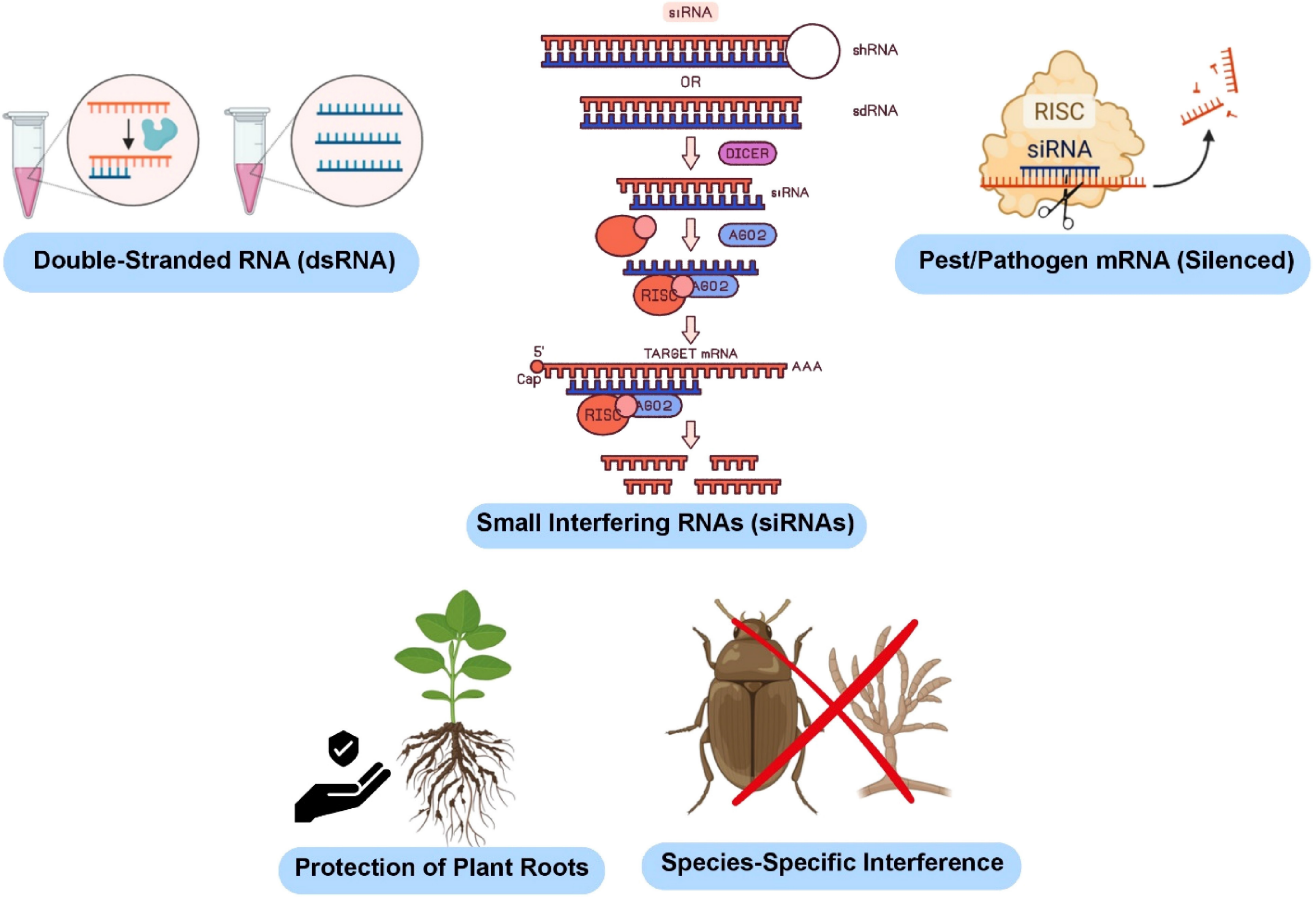
Mechanism of RNA interference (RNAi) for plant protection. dsRNA is processed into siRNAs that guide RISC-mediated cleavage of pest or pathogen mRNA, resulting in species-specific gene silencing and enhanced root protection.

RNA interference (RNAi) and other precision-molecular tools are emerging as “programmable biocontrol” platforms for rhizosphere engineering. In the canonical RNAi pathway, double-stranded RNA (dsRNA) is processed by Dicer into 21-nt small interfering RNAs that guide the RNA-induced silencing complex (RISC) to degrade complementary messenger RNAs, thereby silencing target genes in pests, pathogens, or even the plant host itself (Zhu, 2013; Svoboda, 2020). Figure 4 depicts the RNA interference (RNAi) mechanism for plant protection, illustrating dsRNA processing into siRNAs and RISC-mediated, species-specific silencing of pest or pathogen genes to enhance crop resistance. A landmark case employed an engineered *Escherichia coli* strain that secreted dsRNA targeting the corn pest *Mythimna separata*; feeding larvae the bacterial preparation silenced essential chitinase genes and dramatically reduced feeding damage (Ganbaatar et al., 2017). Similarly, spray-applied dsRNA against *Fusarium graminearum* transcripts conferred disease resistance in barley without transgenic plants (Werner et al., 2020). Host-induced gene silencing (HIGS) extends this concept: transgenic wheat expressing dsRNA against the fungal effector VdAve1 suppressed *Verticillium dahliae* virulence, illustrating how plant-delivered RNAi can reshape the rhizosphere-associated microbiome by disarming pathogens (Song and Thomma, 2018; Maestro-Gaitán et al., 2023).

Advanced molecular approaches integrate RNAi with CRISPR/Cas9 to create “dual-action” microbes (Saffari Natanzi et al., 2025). CRISPR-mediated insertion of dsRNA expression cassettes into beneficial *Pseudomonas* spp. yielded strains that both produce antimicrobial metabolites and deliver RNAi triggers to neighboring pathogens, achieving synergistic disease suppression (Saffari Natanzi et al., 2025). In maize, CRISPR editing of the benzoxazinoid biosynthetic gene altered the root exudate composition, which in turn reprogramed the rhizosphere community to favor beneficial hub taxa and improve herbivore resistance across generations (Kudjordjie et al., 2019). These case studies demonstrate that RNAi delivered via transgenic plants, engineered microbes, or foliar sprays offers a versatile, species-specific means to modulate microbial interactions, suppress pathogens, and steer rhizosphere assembly, positioning RNAi as a cornerstone of next-generation microbiome engineering strategies. Beyond direct suppression of pests and pathogens, RNAi-based approaches may indirectly influence rhizosphere microbial community structure. By modulating pathogen pressure, host immune responses, and root exudate composition, RNAi-mediated regulation can alter microbial competition and niche availability, thereby influencing the recruitment and stability of beneficial microbiome members.

### 3.3 Omics and systems biology

Omics and systems-biology tools are reshaping rhizosphere microbiome engineering by turning descriptive surveys into predictive design platforms (Sinha et al., 2025). Shotgun metagenomics provides a catalog of microbial genes and pathways, enabling researchers to pinpoint functional traits, such as nitrogen fixation, phosphate solubilization, and stress-responsive enzymes, that are enriched in beneficial consortia (Wani et al., 2025). When paired with metatranscriptomics and metaproteomics, these data reveal which genes are actively expressed under field conditions, exposing genotype-by-environment interactions that govern microbiome performance (Wani et al., 2025). Metabolomics complements the genomic view by profiling root exudates and microbial secondary metabolites. For example, untargeted metabolite profiling has identified maize-specific benzoxazinoids that reshape bacterial and fungal community structure, with lasting effects on herbivore resistance across generations (Wang et al., 2024). Single-cell sequencing and fluorescence-activated cell sorting now enable linking individual microbial genomes to their metabolic phenotypes, uncovering rare but keystone taxa that would be missed by bulk analyses (Wu et al., 2023). These integrated metagenomic and metabolomic approaches provide a blueprint for rational design of the rhizosphere microbiome and microbiome-optimized crop breeding.

Functional metagenomic analyses confirmed this convergence at the biochemical level (Dror et al., 2020). Disease suppression was strongly associated with the enrichment of biosynthetic gene clusters (BGCs) encoding non-ribosomal peptide synthetases (NRPS), polyketide synthases (PKS), siderophore biosynthesis pathways, and terpenoid production pathways (Carrión et al., 2019; Tracanna et al., 2021). In wheat, 604 BGCs were significantly overrepresented in suppressive soils by the fifth growth cycle compared to initial conditions (Costa et al., 2023), demonstrating cumulative selection for antimicrobial capacity. In sugar beet, deletion of an *NRPS-PKS* hybrid gene cluster in *Flavobacterium* abolished suppression of *Rhizoctonia solani*, providing direct genetic evidence for its functional role (Pan et al., 2026). Additional enriched BGCs encoded phenazines, polyketides, terpenes, and siderophores (false discovery rate < 0.1), indicating a conserved chemical arsenal underlying pathogen inhibition across crops.

In contrast, microbiomes associated with yield enhancement under low disease pressure were characterized by their dominance in nutrient cycling functions. A 35-year wheat fertilization experiment identified 71 functional genes positively correlated with productivity gains (Fan et al., 2020, 2021). These included carbon fixation genes (*acsA*, *acsB*, *acsE*, *mct*, *rbcl*), nitrogen cycling genes (*ureC*, *hao*, *nifH*, *nosZ1*, *nosZ2*), phosphorus metabolism genes (*phnk*, *phoD*, *phoX*, *ppx*), and sulfur metabolism genes (*yedZ*, *dsrA*). Copy numbers of these genes were highest in keystone taxa exhibiting elevated oxidoreductase activity, linking microbial redox metabolism to crop productivity. Notably, *nifH* abundance was suppressed under long-term NPK fertilization but enhanced by organic matter addition, indicating that inorganic inputs inhibit biological nitrogen fixation (Fan et al., 2021). These functional shifts corresponded with substantial yield increases, including up to 10-fold higher wheat yields in fertilized compared with unfertilized plots, and positive correlations between microbial diversity and potato yield under nutrient-limited conditions (Fan et al., 2019).

Integrative metagenomic and transcriptomic analyses elucidated how microbial functions interface with plant defense and nutrient metabolism (Levy et al., 2018). The phenylpropanoid biosynthesis pathway was consistently enriched in roots grown in suppressive soils, reflecting systemic activation of host immunity. In tomato, soil legacy effects reshaped root transcriptomes, with significant upregulation (*p* < 0.05) of phenylpropanoid pathway genes responsible for flavonoid and salicylic acid biosynthesis. Expression of these genes correlated with bacterial genera including Gp6, *Niastella*, *Actinomarinicola*, *Sphingobium*, and *Sphingomonas*, indicating coordinated plant-microbe signaling (Zhang et al., 2022). In *Arabidopsis*, suppressive taxa such as *Kribbella*, *Nocardioides*, and *Bacillus* activated pathogen-associated molecular pattern-triggered immunity, experimentally confirming microbe-induced systemic resistance (Simmons et al., 2020).

Genes involved in quorum sensing and biofilm formation were also enriched in suppressive systems. In banana soils suppressive to *Fusarium oxysporum* f. sp. *cubense* tropical race 4, overrepresentation of these genes suggested cooperative microbial defense strategies (Shafi et al., 2023). Biofilm formation likely enhances spatial exclusion of pathogens and coordinated antibiotic production (Abebe, 2020). In sugar beet, infection triggered enrichment of genes encoding chitinases, glycoside hydrolases, and glycosyltransferases, providing direct enzymatic mechanisms for fungal cell wall degradation (Wibberg et al., 2016). Similar enrichment of β-glucanase, endoglucanase, and chitinase genes was observed in suppressive wheat soils, indicating a conserved enzymatic defense repertoire (Mapuranga et al., 2022).

### 3.4 Synthetic microbial communities

Synthetic microbial communities (SynComs) and gnotobiotic platforms have become powerful testbeds for rational rhizosphere engineering (Marín et al., 2021). In a gnotobiotic system, plants are grown in sterile substrate inoculated with a defined set of microbes, allowing for the precise attribution of phenotypic effects to individual strains or their interactions (Kremer et al., 2021). Recent perspectives emphasize that successful microbiome engineering requires an ecologically informed design of SynComs, integrating principles such as community assembly, niche complementarity, species interactions, and functional redundancy to enhance the stability and predictability of engineered microbiomes (Henry and Bergelson, 2025). These principles highlight that microbiome engineering should consider ecological processes such as priority effects, environmental filtering, and network stability (Jiao et al., 2022). Incorporating these ecological concepts can improve the persistence and functional reliability of SynComs under field conditions. A seminal study by Bai et al. (2015) employed a root-derived bacterial synthetic community (SynCom) assembled from *Arabidopsis thaliana* isolates to dissect colonization hierarchies; the community reproducibly enhanced shoot biomass and suppressed *Pseudomonas syringae* infection, particularly when *Bacillus subtilis* produced the lipopeptide surfactin, a key determinant of biocontrol and rhizosphere colonization (Bai et al., 2015). Synthetic consortia designed from metagenomic and functional screens have been deployed in field trials. Consortia of phosphate-solubilizing bacteria have been shown to enhance wheat performance, with reported increases of up to 68% in root dry weight and 58% in grain yield (Emami et al., 2020), as well as 8% higher grain yield and 14% greater seed phosphorus content (Yahya et al., 2021). Quorum-sensing (QS) circuitry provides dynamic control over community behavior. Researchers introduced a synthetic LuxI/LuxR system into a rhizobial strain that, upon reaching a density threshold, activates expression of a nitrogenase-optimizing gene cluster, thereby synchronizing nitrogen fixation with the availability of root exudates (Zheng et al., 2006; Vogt et al., 2025). Although direct parallels are limited, studies show strong supporting evidence: expression of the *AiiA* gene, inducible by *Ralstonia solanacearum*, enhanced disease resistance through AHL hydrolysis (Dong et al., 2000; Ouyang and Li, 2016), and engineered strains expressing *AiiA* effectively disrupted pathogen quorum sensing and reduced disease incidence (Cho et al., 2007). These examples illustrate how gnotobiotic assays, functionally curated SynComs, and programmable QS circuits enable predictable manipulation of rhizosphere communities, moving microbiome engineering from empirical inoculation toward a system-level design paradigm.

These emerging technologies are most powerful when integrated into a coordinated microbiome engineering pipeline rather than applied independently. Multi-omics approaches, including genomics, transcriptomics, metaproteomics, and metabolomics, provide foundational insights into microbial diversity, functional genes, and plant–microbe signaling pathways that shape rhizosphere interactions. These datasets increasingly feed into computational frameworks such as genome-scale metabolic models and machine-learning algorithms that predict microbial interactions, community stability, and plant phenotypic outcomes (Ke et al., 2021). The resulting predictions can guide both genome-editing strategies, where CRISPR-based tools modify plant or microbial genes controlling nutrient acquisition, stress responses, or signaling pathways, and synthetic microbial community (SynCom) design, where functionally complementary taxa are assembled according to ecological principles such as niche complementarity, functional redundancy, and keystone taxa effects (Tariq et al., 2025). Through this iterative workflow discovery through multi-omics, prediction through computational modeling, targeted modification via genome editing, and functional deployment through SynCom design microbiome engineering is transitioning from empirical inoculation approaches toward predictive and programmable plant–microbiome systems (Portal-Gonzalez et al., 2025).

### 3.5 Synthetic biology approaches for plant microbiome engineering

Synthetic biology has emerged as a transformative framework for microbiome engineering, enabling the rational design of microbial systems with programmable functions that enhance plant productivity and resilience. Unlike traditional approaches that rely on naturally occurring microbial traits, synthetic biology integrates advanced genetic engineering tools, computational modeling, and ecological design principles to create microorganisms with tailored functions in the rhizosphere. Technologies such as CRISPR/Cas genome editing, phage integrases, and integrative conjugative elements facilitate precise modification of microbial genomes, while genome-scale metabolic models and machine-learning algorithms support predictive design of microbial metabolic networks and community interactions (Ke et al., 2020; Kamath and Patel, 2025).

One major application of synthetic biology involves the engineering of microbial chassis for plant-associated functions. Rhizosphere-adapted bacteria such as *Pseudomonas putida*, *Bacillus* spp., and *Ralstonia* spp. are frequently used as chassis organisms due to their ecological competitiveness, metabolic versatility, and established genetic tractability. These microbes can be engineered to enhance rhizosphere colonization, nutrient cycling, and stress tolerance through targeted modifications of metabolic pathways. For example, synthetic biology strategies have been used to refactor nitrogen-fixation pathways through redesigned nif gene clusters, engineer phosphorus-solubilizing capabilities via phytase expression and citrate secretion pathways, and enhance phytohormone production through optimized auxin biosynthesis systems (Han and Yoshikuni, 2022; Haskett et al., 2021).

In addition to metabolic engineering, synthetic biology enables the construction of modular genetic circuits that allow microbes to respond dynamically to plant and environmental signals. These circuits often integrate quorum-sensing modules, inducible promoters, and transcription-factor-based sensors that detect metabolites released in root exudates. Riboswitch-based regulatory systems and host-specific signaling pathways, such as scyllo-inosamine mediated circuits, can trigger expression of plant growth–promoting traits only under specific rhizosphere conditions. Such programmable circuits enable engineered microbes to function as biosensors capable of monitoring nutrient availability, plant stress signals, or pathogen presence in the soil environment (Portal-Gonzalez et al., 2025; Sharma et al., 2025).

Synthetic biology also facilitates the development of engineered microbial consortia that combine complementary traits across multiple species. By integrating ecological principles such as functional redundancy, niche complementarity, and keystone taxa interactions, synthetic microbial communities (SynComs) can achieve greater stability and performance than single-strain inoculants. These engineered communities have been shown to improve nutrient acquisition, enhance drought tolerance, and suppress plant pathogens in several crop systems, including maize, wheat, and soybean (de Souza et al., 2020). Advances in computational modeling and multi-omics analysis further support the rational design of such communities by predicting metabolic cross-feeding interactions and community stability under variable environmental conditions.

Despite these advances, translating synthetic biology innovations from laboratory systems to field environments remains challenging. Engineered microbes must compete with established native microbiomes and maintain functional stability under fluctuating environmental conditions. Biosafety concerns also necessitate robust containment strategies, including auxotrophic dependencies and environmentally responsive genetic kill-switches, while regulatory frameworks governing genetically modified organisms may limit widespread deployment (Jansson et al., 2023). Consequently, current research increasingly focuses on integrating synthetic biology precision with ecological assembly principles, including the use of native microbial chassis and community-level design strategies. These approaches aim to bridge the gap between laboratory innovation and field-scale agricultural impact, enabling the development of stable, programmable plant–microbiome systems for sustainable and climate-resilient agriculture.

## 4 Microbiome engineering in practice: field-scale evidence and applied innovations

Field-scale evaluations of microbiome engineering strategies have demonstrated that microbial consortia containing 3–21 strains, combined with advanced delivery systems such as mannose nanofibril hydrogels, consistently enhance crop performance, particularly under conditions of abiotic or nutritional stress where native soil microbiomes provide insufficient buffering capacity. In maize, a three-strain consortium (*Pseudomonas* sp. RU47, *Bacillus atrophaeus* ABi03, *Trichoderma harzianum* OMG16) increased shoot dry mass by approximately 30% in field plots in Germany, with consistent effects across both intensive and extensive management systems (Francioli et al., 2025). The inoculant altered the plant’s hormonal balance, enhanced the detoxification of reactive oxygen species, and increased root exudation of phenolic iron-chelating compounds. Metagenomic sequencing revealed enrichment of siderophore, auxin, and quorum-sensing genes in the rhizosphere, providing mechanistic links to the observed drought resilience. Similarly, in tomato, microbial consortium products (MCPs) containing 12 bacterial and fungal taxa outperformed single-strain inoculants under nutrient-limited and stress conditions. In a Romanian greenhouse experiment conducted in low-phosphorus soil with replicated pot trials, yields increased by 39–84%. Under desert field conditions in the Negev, where native soil fertility and phosphorus availability were low, MCPs enhanced fruit biomass by 108% without phosphorus supplementation and by 113–232% when phosphorus was added, highlighting the strong response of microbiome-based interventions under nutrient-limited environments (Bradáčová et al., 2019). The consortia improved phosphate acquisition and shifted rhizosphere composition toward *Flavobacteria*, taxa associated with drought protection.

At the pilot scale, a 21-strain synthetic community (SynCom) assembled from native soils in China increased the maize root:shoot ratio by 78–121% low-fertility soils during replicated greenhouse trials, indicating a strong allocation shift toward root development under nutrient-limited conditions (Jiang et al., 2023). Chlorophyll content rose by 24–35% in Ultisols and 18–27% in Inceptisols, with corresponding increases in plant height increased by 67 and 36% in controlled pot experiments conducted under nutrient-deficient soil conditions, where baseline plant growth was relatively low. The SynCom exhibited superior colonization, stable niche breadth, and reduced competition with the native microbiota, whereas commercial inoculants decreased niche breadth by 59–62%, exacerbating antagonism. This “home-field advantage” underscores the ecological and functional benefits of regionally adapted inoculants over universal commercial strains.

Complementary delivery strategies can sustain microbial activity and plant-microbe contact under environmental stress. Mannose nanofibril hydrogels applied near wheat root zones increased yields by approximately 20% under low-rainfall conditions, correlating with the selective enrichment of beneficial rhizobacteria in hydrogel-extended rhizospheres (Mathes et al., 2020). Metagenomic data indicate that these hydrogels function as continuous wetting pathways, maintaining microbial connectivity and nutrient exchange during desiccation events. A comprehensive meta-analysis of more than 140 experiments under the EU BIOFECTOR program reported a mean 9.3% growth or yield increase across 945 observations, with markedly stronger effects in arid, low-fertility, or drought-stressed systems than in temperate, high-SOM soils (Neumann et al., 2024). Importantly, microbial inoculants performed best when co-applied with manure-based organic fertilizers, which provide carbon sources and micronutrients that promote early root colonization and microbial persistence. Collectively, these data demonstrate that microbiome engineering yields reproducible agronomic benefits primarily when environmental stress exceeds the buffering capacity of native soil communities. Under favorable temperate conditions, abundant indigenous beneficial microbes already supply equivalent ecosystem services, explaining the negligible additional effects observed in German and Swiss field trials (Francioli et al., 2025).

Modern inoculant design employs multi-strain consortia that combine traits such as nitrogen fixation, phosphorus solubilization, and stress mitigation (Sharma et al., 2023). Examples include engineered *Rhizobium* with ACC-deaminase and *Burkholderia-Enterobacter-Pseudomonas* consortia, which enhance nutrient uptake and drought resilience (Kesavardhini et al., 2025). To ensure biosafety, engineered inoculants increasingly incorporate genetic containment systems. Synthetic “kill-switches” trigger cell death under non-target conditions, such as temperatures ≥ 40°C or absence of root-exudate signals, preventing persistence or horizontal gene transfer outside intended rhizospheres (Chan et al., 2016; Varma et al., 2025). This coupling of ecological function with biocontainment reflects a broader maturation of microbiome biotechnology toward precision, safety, and predictability. Applications of microbiome engineering are extending well beyond crop systems into ecological restoration, animal husbandry, and human health. A halotolerant consortium of *Bacillus* and *Enterobacter* consortia applied to saline-affected pastures reduced Na^+^ accumulation and improved soil ionic balance (Shabaan et al., 2022). In metal-contaminated mine tailings, a *Pseudomonas*-*Streptomyces* community reduced cadmium bioavailability by 45% and facilitated the recolonization of native plants (Kondakindi et al., 2024). These cross-domain applications confirm that microbiome engineering, when guided by multi-omics data and ecological principles, can deliver tangible benefits across agricultural, environmental, and biomedical contexts. The convergence of plant genetics, microbial ecology, and synthetic biology enables the rational assembly of microbial ecosystems with predictable functionality. Table 2 depicts outcomes of plant genome editing and synthetic microbiome (SynCom) engineering, highlighting their respective targets, mechanisms, and quantitative impacts on crop performance across diverse agricultural systems.

**TABLE 2:** Comparative outcomes of plant genome editing and microbiome engineering (synthetic community) for crop improvement.

Intervention domain	Trait category/outcome	Edit or engineered system (representative targets)	Organism/ crop	Quantitative or consistent effect	Mode of action/mechanistic basis	References
Plant genome editing	Disease resistance (fungal, oomycete)	Knockout of MLO susceptibility genes (*TaMLO*, *SlMLO1*, *MLO7*) via TALENs/CRISPR	Wheat, tomato, grape	Stable powdery mildew resistance without major yield penalty	Loss of susceptibility factor required for pathogen entry	(Kamburova et al., 2017; Abdelrahman et al., 2021)
	Disease resistance (bacterial blight)	Editing *OsSWEET11*/*13/14* promoters or EBEs	Rice	Durable resistance to *Xanthomonas oryzae* across studies	Disruption of pathogen effector-driven sugar efflux	(Liu et al., 2025)
	Disease resistance (blast)	CRISPR knockout of *OsERF922*	Rice	Enhanced blast resistance; no yield component loss	Removal of negative regulator of immunity	(Vats et al., 2019)
	Broad-spectrum resistance	CRISPR edits in *RBL1*, *OsCUL3a*, *OsPi21*, *OsXa13*	Rice	Resistance to multiple pathogens (*Magnaporthe*, *Xanthomonas*)	Reprograming immune and susceptibility networks	(Kumar et al., 2024)
	Virus resistance	Knockout of *eIF4E*	Cucumber, vegetables	Broad resistance to poty- and ipomoviruses	Disruption of viral translation initiation	(Liu et al., 2025)
	Disease resistance (citrus canker)	Editing *CsLOB1* promoter/CDS	*Citrus* spp.	Strong, reproducible canker resistance	Removal of effector-responsive susceptibility gene	(Miladinovic et al., 2021)
	Yield and grain weight	Editing *GS2*, *GW2*, *GW5*, *TGW6* QTLs	Rice, wheat	+ 10–30% grain size or thousand-grain weight	Direct modulation of yield QTLs	(Abdelrahman et al., 2021)
	Plant architecture	Multiplex editing of domestication genes (*SlCLV3*, *SlWUS*, etc.)	Tomato, wild relatives	∼30% fruit size increase in stacked edits	Control of meristem size and organ number	(Liu et al., 2025)
	Nutrient content	Editing *GGP* genes/*uORFs*	Lettuce, tomato	Up to + 150% vitamin C	Enhanced ascorbate biosynthesis	(Liu et al., 2025)
	Heavy-metal exclusion	Knockout *OsNramp5*	Rice	∼90% reduction in grain Cd	Loss of Cd uptake transporter	(Liu et al., 2025)
	Nitrogen use efficiency	Editing *OsNRT1.1b*	Rice	Improved nitrate uptake under N limitation	Optimized nitrate transport	(Kumar et al., 2024)
	Abiotic stress tolerance	Edits in *DREB1/2*, *ERA1*	Wheat, rice	Improved drought survival with limited yield loss	Stress-responsive TF reprograming	(Altpeter et al., 2016)
Microbiome/ SynCom engineering	N, P nutrition and yield (field)	Multi-strain PGPR SynCom (1,893 isolates screened)	Soybean	Biomass + 4–93%; N + 18–71%; P + 5–68%; yield + 36.1%	Functional assembly (N fixation, P solubilization, hormones)	(Wang et al., 2021)
	Phosphorus uptake	4-strain SynCom (*Burkholderia*–*Pseudomonas* etc.)	Rice	Significant biomass and P uptake increase	P solubilization; Pi transporter upregulation	(Ma et al., 2024)
	Growth and disease suppression	8-strain SynCom (*Bacillus*, *Acinetobacter*, *Enterobacter*, etc.)	Wheat	Increased biomass; reduced *Fusarium* load	IAA, ammonia, antifungal metabolites	(Liu et al., 2022)
	P nutrition vs. fertilizer	3–4 strain P-solubilizing SynComs	Wheat	Outperformed mineral P fertilizer	Organic acids, hormone production	(Mažylytė et al., 2024)
	Growth and yield	4-strain PGPR SynCom (seed coat vs. soil)	Cotton	Yield + 8.5%; soil nitrate + 28–55%	Rhizosphere restructuring; delivery-dependent	(Kaur et al., 2022)
	Root morphology	Multi-PGPR SynCom	Pepper	Shoot height + 21%; fresh weight + 69%	Root system reprograming	(You et al., 2025)
	Disease resistance	5-strain *Bacillus*–*Pseudomonas* SynCom	Pepper	Reduced *Phytophthora* severity; growth increase	Antagonism + N cycling enrichment	(Bashizi et al., 2025)
	Soil health and disease	8-strain *Bacillus*–*Streptomyces* SynCom	Apple	Shoot growth + 5.2%; soil P + 7.9%	Deterministic, disease-suppressive assembly	(Qiao R. et al., 2024)
	Low-N systems	5-strain N-fixing Bacillaceae SynCom	Lettuce	Biomass + 33%	Biological N fixation; inoculation route matters	(Jaoudé et al., 2024)
	Abiotic stress tolerance	15-member SynCom (5 phyla)	*Brachypodium*	Improved drought and salinity recovery	Osmoprotectants; Na^+^/K^+^ transport	(Yadav et al., 2025)
	Wilt resistance	8-strain simplified SynCom (*Pseudomonas*-centered)	Watermelon	Strong *Fusarium* suppression	Hub-taxon synergy; biofilm formation	(Qiao Y. et al., 2024)
	Growth and priming	7-strain SynCom	Tomato	Growth promotion + systemic transcriptional priming	Nutrient mobilization; ISR-like effects	(Montoya et al., 2025)
Cross-system consensus	General PGPR design	*Bacillus*, *Pseudomonas*, *Azospirillum*, *Azotobacter* SynComs	Multiple crops	SynComs outperform single strains	Task sharing; niche complementarity	(Martins et al., 2023)

## 5 Climate-smart microbiome engineering

Climate change increasingly exposes crops to abiotic stresses such as drought, heat, and salinity while simultaneously reshaping soil microbial communities (Benitez-Alfonso et al., 2023). Microbial resilience under these conditions is largely governed by functional traits such as osmolyte production, exopolysaccharide (EPS) synthesis, and spore formation, which enable microorganisms to maintain cellular stability and ecosystem processes during environmental stress (Mouro et al., 2024). These traits are unevenly distributed across microbial taxa and strongly influenced by edaphic factors including soil pH, organic carbon, and clay content (Li et al., 2023). Drought resistance in microbial communities is typically driven by bacterial taxa capable of rapid physiological adjustment. Field and microcosm studies show that Proteobacteria and Actinobacteria maintain community structure during drought and rapidly reactivate nitrogen and carbon cycling after rewetting (Gillespie et al., 2023). In contrast, fungal communities often exhibit lower short-term resistance but may display increased tolerance after repeated drought exposure through physiological acclimation (Dacal et al., 2022; Amarasinghe et al., 2026). Under salinity stress, fungal taxa such as Chytridiomycota and Ascomycota remain metabolically active at NaCl concentrations exceeding 22 mg g^–1^ soil, whereas bacterial communities often decline at similar levels (Rath et al., 2019). Halotolerant plant growth–promoting rhizobacteria (PGPR) including *Bacillus pumilus*, *Halomonas desiderata*, and *Exiguobacterium oxidotolerans* mitigate salinity stress by producing EPS and osmolytes that stabilize soil aggregates and water potential (Bharti et al., 2015).

Thermal stress selects for thermotolerant and spore-forming microorganisms. Bacilli and Actinobacteria frequently increase in abundance under elevated temperatures, with *Bacillus* species surviving transient heat pulses of up to 60°C through endospore formation (Atrih and Foster, 2002). Soil organic matter strongly influences microbial resilience: amended soils maintain higher diversity under heat stress, whereas unamended soils show declines in thermotolerant taxa (Mocali et al., 2022; Ye et al., 2024). Even in amended soils, resistance indices decline from 0.70 after 3 days of heat stress to 0.01 after 28 days, while resilience indices increase from –0.31 to 0.63, indicating that prolonged stress reduces resistance but selects for communities capable of delayed recovery (Lazicki et al., 2025).

At the molecular level, microbial stress tolerance is supported by osmolyte synthesis, EPS production, and sporulation (Malik et al., 2020b). Transcriptomic analyses reveal increased expression of genes involved in trehalose, ectoine, and capsular polysaccharide biosynthesis during drought stress, indicating active physiological adjustment of microbial populations (Cytryn et al., 2007). Synthetic community experiments further demonstrate the ecological relevance of these traits. For instance, a maize rhizosphere consortium enriched in EPS- and osmolyte-producing strains increased seed yield under drought from 9.8 to 38.7 g per plant (Armanhi et al., 2021). However, such stress-tolerance strategies may involve metabolic trade-offs that reduce microbial growth efficiency under non-stress conditions, consistent with the Y–A–S (Yield–Acquisition–Stress tolerance) framework (Radhakrishnan et al., 2017; Malik et al., 2020a).

Network analyses indicate that environmental stress reorganizes microbial interaction patterns. Drought typically weakens positive correlations within bacterial and fungal groups but strengthens cross-domain interactions, particularly between arbuscular mycorrhizal fungi (AMF) and rhizosphere bacteria (Maisnam et al., 2023; Fernando et al., 2023). Although microbial biomass may decline by approximately 31.6% and respiration by 17–65% during stress events, communities frequently recover after disturbance, demonstrating strong ecosystem resilience (Amarasinghe et al., 2026).

### 5.1 Climate-smart microbiome engineering: rhizosphere consortia

Building on these stress-response traits, climate-smart microbiome engineering aims to assemble microbial consortia capable of stabilizing plant performance under environmental stress (Santoyo et al., 2021). The rational design of climate-resilient SynComs increasingly relies on multi-omics datasets to identify strains with complementary metabolic traits and stable ecological interactions (Gonçalves et al., 2023). Functional redundancy and network hub taxa such as *Pseudomonas* facilitate coordinated community behavior through quorum sensing and biofilm formation (Velte et al., 2025). Emerging technologies, including synthetic genetic circuits responsive to environmental cues, may further optimize microbial performance while minimizing metabolic costs under favorable conditions (Guo et al., 2025).

Experimental platforms such as the SoilBox system, capable of simulating 12 cm soil depth, and gnotobiotic plant growth systems enable controlled evaluation of SynCom performance under defined environmental conditions (Bhattacharjee et al., 2020; Kremer et al., 2021). Recent studies demonstrate that engineered microbial consortia can reduce drought-related seedling mortality by 47–71% in Mediterranean tree species and enhance nutrient uptake and hormone regulation in stressed crops (Aleksieienko et al., 2025; Cui et al., 2025). Integration of artificial intelligence and computational modeling may further improve SynCom design by predicting microbial compatibility and field performance under specific environmental scenarios (Tariq et al., 2025).

Overall, climate-smart microbiome engineering integrates microbial ecology, systems-level community design, and advanced biotechnology to develop resilient plant–microbe systems. By combining ecological understanding of stress-tolerant microbial traits with rational SynCom design and emerging computational tools, engineered microbial communities can stabilize crop productivity under environmental stress. Demonstrated yield gains in drought-tolerant maize (∼27%) and salinity-resilient wheat (22%) highlight the potential of these approaches for climate-resilient agriculture (Portal-Gonzalez et al., 2025).

## 6 Integration of microbiome engineering with AI and machine learning

High-throughput metagenomics now provides taxonomic and functional snapshots of the rhizosphere, but translating these data into actionable strategies for microbiome-based crop management requires AI-driven analytics. In a recent field study, shotgun metagenomes and enzyme activity assays were used to train random forests and gradient boosting models, enabling accurate prediction of soil organic carbon dynamics and the identification of microbial taxa that most strongly drive carbon sequestration under variable water regimes (Frey et al., 2022). Concurrently, a hardware prototype that continuously measures pH, moisture, temperature, and NPK concentrations has been coupled with the same machine-learning pipelines; the system achieved over 90% prediction accuracy for real-time soil fertility status and can be extended to forecast microbiome-mediated nutrient availability (Gunasekaran et al., 2025). These approaches illustrate how machine learning can convert complex metagenomic datasets into actionable insights for microbiome-based crop management and rhizosphere engineering. From a microbiome engineering perspective, soil physicochemical properties such as pH, soil organic carbon (SOC), and clay content represent key environmental filters that determine the establishment and stability of engineered microbial communities. These variables influence microbial metabolic activity, nutrient availability, and habitat structure, thereby affecting the colonization success of introduced strains. Consequently, incorporating soil parameters into the design of synthetic microbial communities (SynComs) can improve strain selection for specific soil environments. Integrating these variables into predictive modeling frameworks may also enhance the accuracy of microbiome-based strategies aimed at stabilizing plant performance under climate stress.

Beyond general precision agriculture applications, artificial intelligence is increasingly used to analyze complex microbiome datasets and guide microbiome engineering strategies. Machine-learning approaches can identify microbial taxa associated with plant performance, predict microbial community assembly, and assist in selecting compatible strains for synthetic microbial communities (SynComs). Integrating metagenomic sequencing data with environmental variables further enables predictive modeling of microbiome dynamics and functional outcomes in agricultural systems (Sun et al., 2023; Pace et al., 2025; Miller et al., 2022). These computational frameworks therefore support the rational design of microbiome-based interventions aimed at improving nutrient cycling, disease suppression, and plant stress resilience.

AI-driven modeling has emerged as a transformative approach for integrating microbiome, soil, and climate data to predict agronomic outcomes, yet its effectiveness varies dramatically depending on the prediction target, temporal resolution, and degree of mechanistic alignment among data types (Pace et al., 2025). Reported accuracies range from 13.5% relative root mean square error (RRMSE) for nitrogen loss forecasting to 99.3% classification accuracy for fertilizer and irrigation recommendations, indicating that predictive success depends more on task structure than on algorithmic sophistication (Shahhosseini et al., 2019; Kusumasri et al., 2023; Nyakuri et al., 2022). Tree-based ensemble methods, particularly XGBoost and Random Forest, have consistently outperformed other architectures for structured agronomic data. In contrast, recent transformer-based models demonstrate superior accuracy for short-term decision-making tasks (Natras et al., 2022). Although many AI models focus on yield forecasting, integrating microbiome indicators can improve predictions of disease suppression, nutrient cycling, and plant–microbe symbiosis, which are critical components of microbiome engineering strategies. For crop yield prediction, machine learning models trained on soil and climate variables commonly achieve accuracies between 91% and 97%, with XGBoost emerging as a particularly robust framework (Jain and Srihari, 2022; Kick et al., 2023). Spatially explicit models of maize yield trained on field-scale data achieved up to 95.3% test accuracy and 97.5% area under the curve (AUC) when soil heterogeneity was captured through lattice-based smoothing (Nyéki et al., 2021). When temporal extrapolation rather than spatial interpolation was attempted, predictive error increased sharply, reflecting the inherent uncertainty of long-term, weather-dependent outcomes. The complexity of predicting physiological processes, such as yield, sharply contrasts with that of discrete classification tasks, where transformer-based tabular learning models achieved 99.13% accuracy for irrigation advice and 99.3% accuracy for fertilizer recommendations (Zul Azlan et al., 2024). These findings suggest that model success is constrained less by algorithmic limitations than by the predictability of the underlying biological or environmental process.

Microbiome data have shown particular promise for predicting disease-related outcomes but contribute inconsistently to yield prediction. Machine learning models that integrate microbial community composition with soil physicochemical features explain up to 86% of the variation in plant mycorrhizal growth responses, with fungal community indicators alone contributing 53% of the predictive power and soil properties accounting for 29% (Lutz et al., 2023). The abundance of pathogenic fungi alone explained 33% of the success of arbuscular mycorrhizal fungal (AMF) inoculation, demonstrating that pathogen-symbiont dynamics significantly contribute to microbial influences on plant responses. Similarly, Random Forest models trained on 16S rRNA and ITS sequence data achieved weighted F1 scores of 0.8–0.9 for predicting the occurrence of potato diseases (Aghdam et al., 2024). In contrast, the same datasets performed poorly at predicting yield, supporting the inference that microbial communities exert more immediate and direct effects on disease outcomes than on the multifactorial processes underlying productivity.

The relative contribution of microbiome versus environmental data depends strongly on prediction scope and timescale. Microbiome indicators add substantial predictive value for short-term, biologically mediated phenomena such as pathogen pressure or inoculation response but their utility diminishes for long-term yield prediction, where environmental and management data capture most relevant variance (Ogidi et al., 2024). This asymmetry likely reflects mechanistic proximity, microbial community shifts translate directly into disease incidence but only indirectly into yield outcomes through cumulative effects on nutrient cycling and stress tolerance. Consequently, soil-climate models alone often achieve near-identical performance to integrated microbiome-soil-climate models for yield forecasting, rendering costly microbial sequencing unnecessary for many agronomic applications (Copeland et al., 2025).

The architectures used to integrate heterogeneous datasets generally fall into two categories: feature-level integration, where all data types are concatenated before model training, and model-level integration, where independent submodels generate predictions combined through ensemble averaging or stacking. Feature-level approaches dominate due to simplicity and interpretability. In one example, integrating 48 environmental features, including soil micro-relief, nutrient composition, and meteorological data, into an XGBoost framework allowed non-linear learning of interdependent drivers of yield variability, revealing that K_2_O, NDVI, and soil conductivity were among the strongest positive predictors (Zeraatpisheh et al., 2021). Random Forest models trained with both microbial operational taxonomic unit (OTU) abundances and soil chemistry achieved similar performance when redundant features were removed via stepwise selection, isolating 13 microbial OTUs and 15 soil parameters that together captured over 85% of the variation in plant response (Lutz et al., 2023; Fasolo et al., 2024). Ensemble-level integration, such as combining LASSO, Ridge, Random Forest, and XGBoost models, yielded only modest performance gains of 1–2% over single models (Pardos et al., 2012), suggesting diminishing returns once dominant feature interactions are captured.

The design of synthetic consortia now leverages *in silico* metabolic network reconstruction. Genome-scale metabolic models of candidate rhizosphere isolates were used to score pairwise cross-feeding interactions, and the highest-scoring pairs were experimentally validated in a gnotobiotic platform, demonstrating that AI-guided metabolic modeling is increasingly used to design functionally complementary synthetic microbial communities for crop stress tolerance (Silva-Andrade et al., 2024). Long-read Nanopore sequencing of full-length 16S rRNA genes further refines community profiling, delivering species-level resolution that improves model training and reduces false-positive functional assignments (Aja-Macaya et al., 2025).

These analytical layers are increasingly integrated into smart farming architectures where IoT-connected soil probes, autonomous sampling systems, and cloud-based machine learning platforms enable real-time monitoring of soil microbiomes and environmental conditions (Ambaru et al., 2024). Decision-support algorithms then trigger targeted inoculant applications (e.g., seed-coating dispensers) or adjust irrigation and fertilization schedules to favor beneficial microbes (Rocha et al., 2019). A drought-tolerant maize SynCom, selected through AI-ranked metabolic complementarities, was deployed via drone-mounted sprayers. Sensor-derived moisture maps and on-site metagenomic reads enabled the system to modulate application rates in real-time, resulting in a 22% increase in grain yield in semi-arid plots (Rocha et al., 2019). In a saline wheat trial, IoT-monitored electrical conductivity guided the timed release of a halotolerant *Bacillus* consortium, resulting in a 38% reduction in leaf Na^+^ accumulation and a stabilization of yield across fluctuating salinity spikes (Ilyas et al., 2020). Together, integrated metagenomics, AI/ML predictive modeling, and automated sensing create a feedback loop that transforms rhizosphere microbiome engineering from a static inoculation practice into a dynamic, data-driven component of precision agriculture (Ambaru et al., 2024). Collectively, these advances position artificial intelligence as a critical tool for predictive microbiome engineering, enabling the integration of metagenomic data, environmental variables, and plant performance metrics to guide the design and deployment of beneficial microbial communities.

## 7 Future perspectives: synthetic plant-microbiome holobionts

The next frontier in sustainable agriculture envisions the deliberate co-engineering of plant genomes and their associated microbiomes into adaptive “synthetic holobionts” (Portal-Gonzalez et al., 2025). This concept treats the plant and its microbial partners as a single, heritable biological system capable of coordinated adaptation to environmental stress (Hassani et al., 2018). While heritable, host-mediated control over microbiome assembly has been repeatedly documented, and synthetic microbial communities (SynComs) have demonstrated reproducible benefits under controlled conditions (Ali et al., 2024). The available evidence, including the work by Mitter et al. (2017) on *Paraburkholderia phytofirmans* PsJN colonization of wheat, confirms that stable vertical transmission of beneficial microbes through seeds is achievable without altering host genomes. In that study, flower inoculation established a seed-borne bacterial lineage that recovered after 12 months of storage, resulting in earlier spike onset and greater ear density per square meter. However, this represented microbiome modification alone; coordinated manipulation of plant and microbial genomes in concert, a true synthetic holobiont, remains a theoretical but compelling goal.

Transgenerational inheritance further supports the idea of plants and microbes as an integrated evolutionary unit. Seed-borne endophytes facilitate the vertical transmission of microbial consortia, thereby forming a stable “microbial memory” that spans generations (Truyens et al., 2015). Multiple studies demonstrate that stress-induced parental and microbiome-mediated effects on plant fitness are common, genotype-specific, and non-linear, with offspring performance varying from strong negative to positive responses depending on both ancestral and current environments (Latzel et al., 2023; Groot et al., 2016). Disruption of soil microbiomes can markedly alter stress responses, particularly root traits under drought, and cryptic microbial effects may reshape plant selection across generations (Boyle et al., 2024). Yet, translating these findings into practical, field-scalable co-engineering remains challenging due to the inherent instability of microbiome assembly and the high context-dependence of plant–microbe interactions (Morales Moreira et al., 2023). Emerging biotechnologies now make the construction of the synthetic holobiont conceptually feasible. On the plant side, genome-editing tools such as CRISPR–Cas9 enable precise modification of genes governing microbial recruitment and signaling (Nandavaram, 2024). Edits targeting root-exudate biosynthesis pathways or immune receptor families could enhance the attraction of beneficial taxa or tolerance to specific microbial colonists. For instance, benzoxazinoid-deficient maize mutants have been shown to restructure bacterial and fungal assemblages, indirectly improving herbivore resistance across generations (Hu et al., 2018). Meanwhile, maize landraces that exude sugar-rich mucilage from their aerial roots naturally assemble nitrogen-fixing consortia, offering a genomic blueprint for designed holobionts (Higdon et al., 2020). In parallel, microbial genome engineering enables the direct insertion of beneficial traits into inoculant strains. Nitrogen-fixation pathways, stress-response operons, and biosynthetic gene clusters can be incorporated to enhance resilience and plant growth (Ke et al., 2021). CRISPR-mediated edits and RNA-interference circuits have been used to boost phosphate solubilization, reduce ethylene synthesis via ACC-deaminase, and integrate environmental “memory modules” that modulate microbial behavior under stress (Wang et al., 2017). Biosafety safeguards, such as toxin–antitoxin or nutrient-dependent kill switches that activate when root exudates are absent, are being developed to prevent the escape of engineered microbes into non-target ecosystems (Halvorsen et al., 2022).

Synthetic microbial consortia provide an applied foundation for this dual-engineering vision. Multi-strain SynComs derived from metagenomic screens of drought- or salinity-adapted rhizospheres routinely outperform single isolates, due to functional redundancy and synergistic interactions (Dubey et al., 2025). SynCom treatments could increase maize yield from 11 to 16 tons/ha in field conditions. Similarly, SynComs assembled from sugarcane or wheat rhizospheres have demonstrated improved drought and salinity tolerance, confirming that microbial community design can influence crop performance under field stress conditions (De la Vega-Camarillo et al., 2023). Pawlowski et al. (2020) identified six quantitative trait loci in soybean that together accounted for approximately 24% of the phenotypic variance in mycorrhizal colonization, and pinpointed candidate nodulin protein families and symbiosis-specific genes as promising targets for genetic manipulation.

Despite this promise, achieving truly integrated, stable plant-microbiome systems faces substantial obstacles. The ecological stability of engineered consortia remains uncertain across soil types and climatic gradients; introduced microbes often lose competitiveness against indigenous microbiota. Evolutionary divergence between host and symbiont genomes can initially decouple beneficial interactions, and regulatory frameworks for genetically modified holobionts remain underdeveloped. Additional challenges include large-scale production and formulation of multi-strain inoculants, ensuring consistent colonization, and addressing societal concerns regarding engineered symbioses (Schmitz et al., 2022). Overcoming these barriers will require coupling synthetic biology with predictive ecological modeling, long-term field trials, and participatory design involving agronomists and farmers. Multi-omics data integration combining host genomics, metagenomics, transcriptomics, and metabolomics will be essential to identify stable plant-microbe gene networks that can be co-optimized through machine-learning-guided design.

## 8 Conclusion

Plant microbiome engineering has rapidly evolved from empirical inoculation practices to a precision-driven discipline that integrates molecular genetics, microbial ecology, systems biology, and digital analytics. This transition reflects a deeper recognition of the plant and its associated microbial community as a single functional unit, the holobiont, whose collective genome, or hologenome, governs plant health, resilience, and productivity. Traditional strategies such as microbial inoculation, crop rotation, and organic amendments have yielded important insights into soil–plant–microbe interactions, yet their effectiveness is often constrained by environmental variability, inconsistent field performance, and limited microbial persistence. The emergence of CRISPR/Cas genome editing, RNA interference (RNAi), and synthetic microbial communities (SynComs) has introduced unprecedented precision in reshaping plant–microbiome interactions. Targeted editing of plant and microbial genomes enables manipulation of genes controlling nutrient acquisition, immune signaling, and stress adaptation, thereby enhancing nitrogen fixation, phosphorus solubilization, and disease resistance with greater predictability. When combined with RNAi-based strategies for silencing pest and pathogen genes, these tools expand the capacity to design environmentally compatible and durable microbiome interventions. Parallel advances in multi-omics platforms, including metagenomics, transcriptomics, and metabolomics, have revealed the functional complexity of the rhizosphere and provided a rational basis for assembling crop and environment-specific SynComs. Increasingly, artificial intelligence (AI) and machine learning (ML) frameworks are bridging the gap between microbiome data and field-level decision-making. By integrating microbiome profiles with soil, climate, and management variables, AI-enabled models facilitate the prediction of biologically proximal outcomes such as disease risk, inoculant responsiveness, and stress sensitivity. Importantly, these tools support context-aware and selective deployment of microbiome-based interventions rather than universal, data-intensive approaches, reinforcing the need for biologically informed model design. As part of precision agriculture systems, AI-driven analytics enable adaptive feedback loops in which microbiome composition, environmental signals, and agronomic practices are dynamically aligned. Looking ahead, the next generation of plant microbiome engineering will focus on climate-smart, self-regulating microbial consortia capable of maintaining soil fertility and crop performance under drought, salinity, and temperature extremes, while reducing reliance on synthetic fertilizers and pesticides. Realizing this potential requires that technological innovation be accompanied by rigorous biosafety frameworks, ecological risk assessment, and transparent governance to address concerns related to horizontal gene transfer, ecological disruption, and societal acceptance. Ultimately, the convergence of genome editing, synthetic biology, multi-omics, and AI-driven precision agriculture positions plant microbiome engineering as a cornerstone of sustainable, climate-resilient, and equitable food systems. Achieving this vision will depend on interdisciplinary collaboration among molecular biologists, soil ecologists, data scientists, agronomists, and policymakers, ensuring that microbiome-based innovations translate from experimental promise to reliable and responsible agricultural practice in the twenty-first century.
